# Bisphosphonates as disease-modifying drugs in osteoarthritis preclinical studies: a systematic review from 2000 to 2020

**DOI:** 10.1186/s13075-021-02446-6

**Published:** 2021-02-22

**Authors:** Silvia Fernández-Martín, Mónica López-Peña, Fernando Muñoz, María Permuy, Antonio González-Cantalapiedra

**Affiliations:** Anatomy, Animal Production and Veterinary Clinical Sciences Department, Veterinary Faculty, Universidade de Santiago de Compostela, Campus Universitario s/n, 27002 Lugo, Spain

**Keywords:** Osteoarthritis, Bisphosphonates, Disease-modifying drugs, Animal models, Subchondral bone, Biochemical markers

## Abstract

Bisphosphonates have been proposed as possible disease-modifying drugs in osteoarthritis. However, the evidence of their efficacy is poor and their outcomes presented a great heterogeneity. Therefore, the aim of this study is to systematically review the main effects of bisphosphonate use on synovial joint tissues and biochemical markers in preclinical studies over the past two decades (2000–2020). Three databases (Pubmed, Scopus, and Web of Science) were searched, and after screening, twenty-six studies with five different types of bisphosphonates were included in the review. The animal model selected, the type of bisphosphonate used, the therapy duration, and the main effects of individual drugs on synovial tissues were evaluated. Additionally, the quality and risk of bias assessments were performed using the Animals in Research Reporting In Vivo Experiments guidelines and the Systematic Review Centre for Laboratory animal Experimentation tool. Studies showed high variability in experimental designs. Consequently, the comparison of the findings in order to draw specific conclusions about the effectiveness of the drugs is complicated. However, the results of this systematic review suggested that bisphosphonates seemed to reduce the osteoarthritic changes in a dose-dependent manner showing better chondroprotective effects at high doses. Besides, a time-dependent efficacy was also detected in terms of cartilage status. One can conclude that the disease stage of the time-point of treatment initiation may constitute a key factor in the antiresorptive drug efficacy. Generally, we noted that bisphosphonate administration seemed to show positive subchondral bone conservation and fewer biomarker alterations. However, they did not appear to suppress the osteophyte development and their chondroprotective effect is highly variable among the studies. Bisphosphonates appeared to show a positive anti-inflammatory effect on the synovial membrane. However, only a few included publications were focused on their investigation. Regarding the therapy duration, there is a significant lack of evidence on evaluating their effectiveness in preclinical long-term studies and further experimental studies may be needed to examine the pharmacological response in these circumstances. This systematic review might help to clarify the efficacy of bisphosphonates and their function as disease-modifying treatments in osteoarthritis.

## Introduction

Osteoarthritis (OA) is thought to be the most prevalent chronic degenerative joint disease in animals and humans, leading to pain, stiffness, and disability. Structural changes include osteophyte formation, synovial inflammation, bone remodeling, and pathological changes in cartilage and menisci, among others [1]. The physical impact and the economic burden of this musculoskeletal disease are immense, affecting approximately 15% of the human global population (> 50% of the aging population over 60 years of age) [2, 3] and being ranked the 12th major cause of global disability in 2016 [4]. Regarding veterinary medicine, it is highly prevalent in dogs and causes noticeable signs of pain and lameness [5]. However, the current information on the prevalence of canine OA is limited and the reported values are widely variable [6]. Additionally, contrary to humans, there is little epidemiological data available on this disease in the different animal species [7].

At present, there are no identifiable disease-modifying treatments for OA; thus, its management is an enormous challenge. Scientific community has been working for years on the development of systemic drugs, which can slow down or prevent the articular cartilage damage and subchondral bone changes, as well as reduce the pain and other symptoms. It has been suggested that the status of the subchondral bone compartment should be a potential target in OA [8, 9]. In relation to this, antiresorptive drugs have been studied for their possible beneficial effects on decreasing bone remodeling and improving bone mineralization and trabecular microarchitecture. Within the available options, bisphosphonates (BPs) have been considered to have a positive impact on articular cartilage and periarticular bone changes by inhibiting bone-resorption activity [10]. However, their mechanism of action and effectiveness in OA is not yet clear.

To better understand the pathophysiology and evolution of this illness, many animal models have been developed as an attempt to mimic the natural human disease. The in vivo preclinical animal studies play a key role in the study of the therapeutic drug efficacy, allowing the histopathological analysis of affected joints at different disease stages [11, 12]. Additionally, recent advances in diagnostic techniques, such as new imaging modalities and biochemical assessments, have proven useful to improve our understanding of the disease, allowing us to evaluate all essential joint tissue components [11]. As previously mentioned, BPs have been proposed as possible disease-modifying drugs in OA. However, both in preclinical and clinical studies, research findings are inconsistent. Some systematic reviews of randomized controlled trials were conducted as an attempt to clarify the effect of BPs in human OA. All of them were in agreement that there was an important heterogeneity across the studies analyzed. They mainly concluded that BPs showed limited evidence for pain control or symptomatic clinical relief, and additionally, no radiographic changes were observed [13–15]. Regarding experimental preclinical studies with animal models, there are currently no updated systematic reviews evaluating the impact of BPs on structural OA changes. Consequently, we consider it may be of interest to elucidate their function as possible disease-modifying treatments.

This systematic review will overview the efficacy of commonly used bisphosphonates for OA treatment in experimental animal models. The aim of this study is to record and categorize the outcome measures on synovial joint tissues and biochemical markers in preclinical studies by systematically reviewing the last two decades of peer-reviewed publications on OA.

## Methods

### Protocol and search strategy

This systematic review was conducted and reported according to the formal PRISMA guidelines (“Preferred Reporting Items for Systematic Reviews and Meta-Analyses”) [16, 17]. The search strategy was performed in the following online databases: PubMed, Scopus, and Web of Science (WOS). The studies were identified using the combination terms: “osteoarthritis,” “bisphosphonates,” and “animal models” as keywords.

### Inclusion and exclusion criteria

The inclusion criteria were as follows:

- Experimental studies in animal models of OA in which the effect of bisphosphonate drugs on biochemical markers and knee synovial joint tissues such as cartilage, synovial membrane, and subchondral bone were assessed, through gross, histology, biochemical, and/or imaging techniques.
- Studies published in internationally peer-reviewed journals between 2000 and June 2020.
- Articles published in English.
- Accessible by authors through Internet searching or institutional access.

The exclusion criteria were articles written in other languages, reviews, book chapters, in vitro studies, clinical trials, and reports in which none of the outcomes of interest were analyzed.

### Study selection and data extraction

At first, titles and abstracts were selected through an online search for inclusion. Next, the screening process was conducted using the inclusion and exclusion criteria. Subsequently, the full text of articles assessed for eligibility was screened and duplicates were removed. Assessments were performed by a single author (SFM) with team consensus by all authors. The authors extracted the following information from each paper included: animal characteristics (species, gender, and age), number of animals, osteoarthritis animal model, drug therapy (dosage, frequency, and duration), baseline, methods of evaluation, and main results. The outcome measures reported in each publication were recorded and categorized for comparison.

Additionally, the studies were grouped according to the duration of the treatments, into short-term treatments (≤2 months), intermediate-term treatments (between 2 and 6 months), and long-term treatments (≥6 months). In studies where the drug administration was made on different specific days, we selected the longest term.

Lastly, we performed a qualitative synthesis of the main findings, summarizing the effects of the BPs evaluated on structural joint tissues (cartilage, subchondral bone, and synovial membrane), osteophyte development, and biochemical markers. We classified the outcomes as positive effect (+), negative effect or no effect (−), and unclear effect (?). The latter was determined when contradictory outcomes were observed or when we had an initial positive response, but not maintainable over time. Also, we marked as not included (×) when the parameters were not evaluated.

### Risk of bias and quality assessments

Two independent authors (SFM and AGC) performed the quality and risk of bias assessments, and any discrepancies were resolved with team consensus by all authors. We systematically analyzed the quality of the included in vivo preclinical studies using the Animals in Research Reporting In Vivo Experiments (ARRIVE) guidelines [18]. For this purpose, we checked each item of the 20 included in this checklist and responded with “yes” if the publication complies, “no” if it does not, and “unclear” if the details were not completed in all sub-items.

The risk of bias was assessed using the Systematic Review Centre for Laboratory animal Experimentation (SYRCLE) tool for animal studies [19], an adapted version of the Cochrane Risk of Bias tool for randomized controlled trials, where additional criteria specific to animal studies were added. The risk-of-bias tool contains 10 entries with specific signaling questions. In order to assign a judgment of low, high, or unclear risk of bias to each item, when responding the signaling questions with “yes” indicated low risk of bias, “no” indicated high risk of bias and “unclear” if insufficient details were reported to assess the risk of bias properly. The entries were classified as high risk of bias if one or more signaling questions were not met and at unclear risk of bias if one or more were partly satisfied.

## Results

### Study selection

**Fig. 1:**
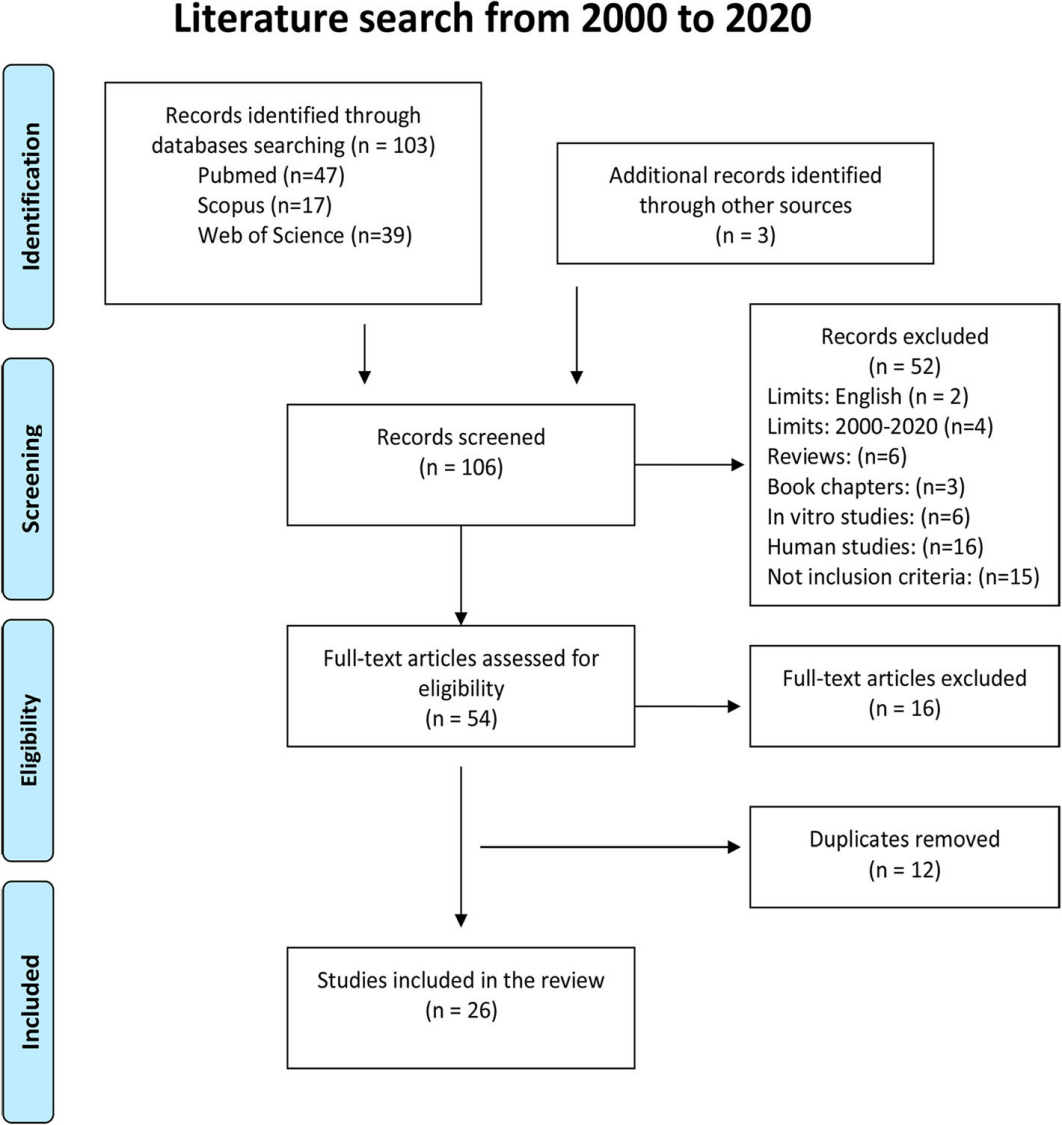
Search strategy according to Preferred Reporting Items for Systematic Reviews and Meta-Analyses (PRISMA) guidelines

The literature search resulted in 103 potentially eligible publications, identified and screened in the initial search. Forty-seven articles were retrieved using PubMed www.pubmed.com, 17 articles using Scopus www.scopus.com, and 39 articles using Web of Science www.webofscience.com. In addition, 3 articles were identified through other resources. The remaining publications (*n* = 106) were screened, and after the evaluation of the inclusion and exclusion criteria, 52 records were excluded. After screening, 54 individual studies were identified as potentially eligible and checked in full-text. Out of them, 16 full-text articles were excluded and 12 duplicate articles were removed. Finally, a total of 26 articles were found suitable to be included in the present systematic review. They dated from 2002 to 2017 and were found in 13 journals. The study selection process can be found in Fig. 1.

### Study characteristics and results of individual studies

Main data extraction from the included articles is shown in Table 1. The results are explained below in detail, based on the animal model selected, the type of bisphosphonate used, the therapy duration, and finally, the main drug effect observed.

**Table 1:** Characteristics of included preclinical studies

References	Animal model (n)	OA model and site	Therapy: dosage, frequency and duration	Start point *	Follow-up and evaluations	Main results
She et al. [20]	New Zealand Rabbit; Male 5–7 monthsold (32)	Surgically-induced OA: ACLT Knee	Zoledronic acid (250, 50 and 10 μg/kg i.v.) Once post-surgery	0 days	0, 4, and 8 weeks X-ray absorptiometry scanner (BMD) and MRI (cartilage thickness). Histology of cartilage (Mankin score system)	ZOL improved the microstructure and reduced the degeneration of articular cartilage in a dose-dependent manner, showing better chondroprotective effects at the high doses. On subchondral bone, ZOL ↑BMD.
Lampropoulou-Adamidou et al. [21]	New Zealand Rabbit; Male 25 weeksold (18)	Surgically induced OA: ACLT Knee	Zoledronic acid (0.6 mg/kg i.v.) On day 1, 15, and 29	1 day	8 weeks Macroscopic evaluation Histology of cartilage (modified Mankin score)	Macroscopically ZOL group had significantly milder ulcerations, cartilage softening and fibrillation. Microscopically ZOL showed chondroprotective effect.
Permuy et al. [22]	New Zealand Rabbit; Female 6–7 months old (32)	Surgically induced OA: ACLT and partial MMT. Knee	Glucosamine (21.5 mg/kg/day oral) +/or Risedronate (0.07 mg/kg/day oral) 8 weeks	3 weeks	11 weeks Histology of cartilage and synovial (OARSI score). Histomorphometric evaluation and μ-CT (BV/TV, Tb.Th, Tb.Sp, Tb.N, Tb.Pf)	RIS treatment alone or in combination showed improves cartilage swelling, superficial fibrillation and less inflammatory changes in the synovial membrane. On subchondral bone, RIS modify the orientation of trabecular lattice (↓Tb.Sp and ↑Tb.N)
Shirai et al. [23]	New Zealand Rabbit; Female 9 months old (30)	Surgically induced OA: ACLT Knee	Alendronate (0.14 mg/kg/weekly s.c.) 2, 4 or 12 weeks	0 days	2, 4, or 12 weeks Histology of cartilage (Mankin score system) μ-CT (BV/TV), subchondral bone plate thickness and osteophyte development. Immunohistochemistry (MMP13, IL-1β, COLX, VEGF and RANKL)	ALN showed chondroprotective effect and prevented periarticular bone loss. Immunohistochemical analysis showed that ALN suppressed the expression of MMP13, IL-1β, COLX, VEGF, and RANKL in OA cartilage.
Zhang et al. [24]	Japanese Rabbit; Male 10 weeks old (30)	Surgically-induced OA: ACLT Knee	Alendronate (10 μg/kg/day s.c.) 56 days	4 days	60 days Histology of cartilage (Mankin score system) X-ray absorptiometry scanner (BMD) and histomorphometric assays (BV/TV, Tb.Th, Tb.N, Tb.Sp). Immunohistochemistry (BMP-2 and MMP-13)	ALN treatment reduced cartilage lesions and delayed the cartilage degeneration. Significantly suppressed subchondral bone resorption (↑BMD, BV/TV, Tb.Th, and Tb.N). Also, showed chondroprotective role in immunohistochemistry assays (↑BMP-2 and (↓MMP-13).
MacNeil et al. [25]	New Zealand Rabbit; Female Mature (18)	Surgically-induced OA: ACLT Knee	Risedronate (0.01 mg/kg/day s.c.) 6 weeks	?	6 weeks μ-CT (BV/TV, BS/BV, Tb.N, Tb.Th, Tb.Sp, Conn.D, Ct.Th, BMD)	RIS group exhibited areas of developing osteophytes. Cartilage surface showed only focal roughening. RIS animals showed periarticular bone conservation (BV/TV, Ct.Th)
Doschak et al. [26]	New Zealand Rabbit 1 year old (28)	Surgically-induced OA: ACLT Knee	Risedronate (0.01 mg/kg/daily s.c.) 6 weeks	?	6 or 14 weeks Histology of cartilage (Modified Mankin score system) μ-CT (laxity and volume of the MCL)	RIS treatment conserved periarticular bone and improved MCL-complex laxity. However, showed the higher scores on the modified Mankin scale.
Doschack et al. [27]	New Zealand Rabbit 1 year old (30)	Surgically induced OA: ACLT Knee	Risedronate (0,01 mg/kg/daily s.c.) 6 weeks	?	6 weeks Histology of cartilage (Modified Mankin score system). X-ray absorptiometry scanner (BMD) Biochemical of periarticular bone	RIS treatment conserved periarticular BMD and ligament mechanical porperties. RIS did not have de capacity to supress osteophytosis nor early cartilage signs of degradation.
Muehleman et al. [28]	New Zealand Rabbit; Male Adolescent (58)	Chemically induced OA: Chymopapain Knee	Zoledronic acid (10 μg/kg/3 times per week s.c.) 8, 28, or 56 days	1 day	28 or 56 days Macroscopic evaluation Histology of cartilage and PG content Urine samples (collagen cross-links)	ZOL treated animals displayed a significantly lower degree of grossly and histologically cartilage degeneration. Urinary levels of collagen cross-links were higher in untreated animals.
Cinar et al. [29]	Wistar Rat Male Adult (48)	Surgically induced OA: ACLT Knee	Zoledronic acid (10 μg/kg/weekly intra-articular) 4 days, 3 or 6 weeks	0 days	4 day, 3 weeks, or 6 weeks Histology score of cartilage (Mankin score system) and synovial Serum analyses (COMP)	ZOL intra-articular administration showed significant reduced synovitis and partially chondroprotective effect, although did not completely prevent cartilage damage.
Bagi et al. [30]	Lewis Rat Male 4 month old (48)	Surgically induced OA: MM Knee	Zoledronic acid (100 μg/kg/2 times per week s.c.) PTH (40 μg/kg/5 times per week s.c.) 10 weeks	0 days	5 and 10 weeks Histological score (OARSI adapted scale) μ-CT (BV/TV, BS/BV, Tb.N, Tb.Th, Tb.Sp) Serum analyses (Osteocalcin, P1NP, CTX-1, CTX-II)	ZOL and PTH improved subchondral bone mas (↑BV/TV, TB.N and Tb.Th), but both treatments failed to prevent or correct cartilage deterioration, thickening of the subchondral bone plate, osteophyte formation nor the mechanical incapacity. ZOL ↓CTX-II level serum.
Zhu et al. [31]	Sprague-Dawley Rat; Female 7 months-old (78)	Spontaneusly model: Menopause-OA (OVX) Knee	Alendronate (30 μg/kg/twice weekly s.c.) 2, 10 or 18 weeks	0 or 8 weeks	2, 10, or 18 weeks Histology of cartilage (Mankin score system) μ-CT (BV/TV, Tb.Th, Tb.N, Tb.Sp) Urinary CTX-I and serum CTX-II. Inmunohistochemistry (MMP-9, MMP-13)	Early ALN treatment prevented both subchondral bone loss and cartilage surface erosion. Late ALN treatment was able to inhibit subchondral bone loss but did not reverse cartilage erosion. ALN ↓MMP-13 and MMP-9.
Mohan et al. [32]	Wistar Rat Male 8 weeks-old (84)	Chemically induced OA: MIA Knee	Alendronate (15 μg/kg/2 times per week s.c.) Pre-emptive: day 0 to 14; early: day 14 to 42; delayed: day 42 to end of week 10	0, 14, or 42 days	2, 6 and 10 weeks Histology score (OARSI scoring system) μ-CT (BV, BV/TV, Tb.Th, Tb.Sp, and Tb.N) Serum analyses (COMP, CTX-I) and urine CTX-II	Pre-emptive ALN treatment preserved subchondral bone, decreased bone turnover and had moderate effects on cartilage degradation. Early and delayed ALN treatments prevented bone loss and decreased bone turnover, but had no significant effect on cartilage degradation.
Panahafir et al. [33]	Sprague-Dawley Rat; Female 6 weeks-old (15)	Surgically induced OA: MMT Knee	Alendronate (0.12 mg/kg/twice weekly s.c.) 8 weeks	1 day	0, 4, and 8 weeks Histologic assessments of cartilage (Modified Mankin score system) μ-CT (BMD and BV of osteophytes)	ALN treatment inhibited osteophyte development and were more cartilaginous (↓BMD). Also, ALN showed reduced degeneration of the cartilage.
Koh et al. [34]	Sprague-Dawley Rat; Female 6 months-old (30)	Chemically induced OA: MIA Knee	Pamidronate (3 mg/kg/weekly s.c.) 8 weeks	8 weeks earlier	8 weeks after induced OA Macroscopic evaluation (ICRS grading system) μ-CT (BV/TV, Tb.N, Tb.Th, Tb.Sp, Tb.Pf, SMI)	PAM treatment showed less trabecular bone changes and cartilage damage
Jones et al. [35]	Sprague-Dawley Rat; Female 6 weeks old (58)	Surgically induced OA: KTI Knee	Alendronate (0.12 mg/kg/2 times per week s.c.) Risedronate (0.06 mg/kg/2 times per week s.c.)	1 day	1 day, 4 and 8 weeks Histologic assessments (Modified Mankin scoring system on toluidine blue and safranin-O stained samples) MRI and μ-CT (BV and osteophyte formation)	Treatments with BPs showed reduced levels of trabecular bone loss (↑BV). ALN reduced bony osteophyte development, but RIS did not positively impact. Histologic analysis confirmed the chondroprotective effect of both BPs.
Strassle et al. [36]	Sprague-Dawley Rat; Male 8 weeks-old (195)	Chemically induced OA: MIA Knee	Zoledronic acid (10, 30, or 100 μg/kg/every third day s.c.) Pre-emptive: day 1 to 21; early: day 14 to 21 or 35; delayed chronic: day 21 to 35; sub-chronic: 28 to 35	1, 14, 21, or 28 days	5 or 22 days X-ray absorptiometry scanner (BMD) Histological analysis (toluidine blue and TRAP stained samples)	Pre-emptive ZOL treatment ↑BMD and prevented the thinning of the cartilage, loss of proteoglycan and chondrocytes. Also, cartilage and subchondral bone resorption. In advanced OA, ZOL partially restored BMD.
Hayami et al. [37]	Sprague-Dawley Rat; Male 20 weeks-old (95)	Surgically induced OA: ACLT Knee	Alendronate (15 or 120 μg/kg/2 times per week s.c.) 2 or 10 weeks	3 days	2 or 10 weeks Macroscopic evaluation and histological analysis (Modified Mankin score system) Histomorphometric assays: subchondral bone volume (BV/TV) and osteophytes. Serum and urinary analyses (COMP, CTX-I, CTX-II) Inmunohistochemistry (MMP-13, MMP-9, TGFβ)	ALN showed chondroprotective effect, suppressed subchondral bone resorption and reduced osteophyte area (dose-dependent manner). ↓MMP-13, MMP-9 and TGFβ.
Adebayo et al. [38]	C57BL/6 (B6) and FVB/NJ (FVB) Mice Male 26 week old	Non-invasive loading OA: CACTC Knee	Alendronate (26 μg/kg/day i.p.) 1, 2 or 6 weeks	0 days	1, 2, or 6 weeks Histology of cartilage and osteophyte (Modified OARSI scoring system)μ-CT (BV/TV, Tb.Th, Tb.Sp)	ALN treatment inhibited bone remodeling and, in B6 mice cartilage pathology was exacerbated, while in FVB mice cartilage loss was protected. ALN inhibited osteophyte maturation, but did not affect osteophyte size.
Khorasani et al. [39]	C57BL/6 N Mice Female 10 weeks old (90)	Non-invasive loading OA: Tibial compression overload Knee	Alendronate (40 or 100 μg/kg/twice weekly s.c.) 7, 14 or 56 days	0 days	7, 14 or 56 days Histologic assessments (OARSI score system) μ-CT (BV/TV, Tb.Th, BMD, Ct.Th, and osteophyte volume) Serum analyses (CTX-I and P1NP)	High-dose ALN of treatment was able to prevent early trabecular bone loss and cartilage degeneration, but was not able to inhibit osteophyte formation, nor was it able to mitigate long-term degeneration. ALN ↓CTX-I serum
Sniekers et al. [40]	C3H/HeJ Mice Female 12 weeks old (32)	Menopause-related OA (OVX) and chemically-induced OA: MIA Knee	Estradiol (12 μg/day s.c. implant) Alendronate (2 mg/kg/weekly i.p.) 12 weeks	0 days	12 weeks Histology of cartilage and osteophyte (OARSI score system) μ-CT (Subchondral cortical thickness, BV/TV, osteophytosis)	ALN ↑subchondral cortical bone thickness and BV/TV and tended to diminish cartilage damage.
Thomsen et al. [41]	Dunkin Hartley Guinea Pig Male 3 months old (56)	Spontaneusly model: Naturally occurring Knee	Risedronate (30 μg/kg/five times a week s.c.) 6, 12, or 24 weeks	–	0, 6, 12, or 24 weeks Histologic assessments (OARSI score) and histomorphometry. Indentation mechanical testing (subchondral bone plate stiffness). Serum CTX-II	RIS did not reduce the articular cartilage damage and did not influence on subchondral bone plate stiffness, but ↓ serum CTX-II. RIS treatment reduced bone resorption and bone formation.
Ding et al. [42]	Dunkin Hartley Guinea Pig Male 6.5 months old (66)	Spontaneusly model: Naturally occurring Knee	Alendronate (10 or 50 μg/kg/twice weekly s.c.) 9 or 17 weeks	–	9 or 17 weeks Histology score of cartilage (Mankin scoring system on safranin-O samples-CT (Subchondral bone plate thickness, Tb.Th, Tb.Sp, and BS/BV) Bone density and mineral	ALN groups showed worse cartilage degeneration in spite of subchondral bone plate thickness, bone mineral content and density.
Dearmin et al. [43]	Mixed-breed Dog; Male 11–24 months old (24)	Surgically induced OA: ACLT Knee	Zoledronic acid (10 or 25 μg/kg/every 3 months s.c.) 12 months	1 day	0, 1, 6, 9, and 12 months Serum, synovial and radiographic analysis (BAP, type I and II collagen, CS846) Gross articular analyses of cartilage, meniscus and osteophyte lesions	ZOL high-dose group resulted in a chondroprotective effect with lower articular defects but did not have the capacity to prevent osteophytosis nor the progression of the radiographic lesions. In synovial fluid, ZOL ↓type I and II collagen.
Pelletier et al. [44]	Crossbred dog - 1–4 years old (31)	Surgically induced OA: ACLT Knee	Tiludronic acid (2 mg/kg on days 14, 28, 56 and 84 s.c.) + extracapsular stabilization surgery	14 days	1 year Macroscopic evaluation and histological assessments of cartilage and synovial (Modified Sakakibara scale). MRI and cartilage volume PCR (ADAMTS-4 and 5, MMP-1, MMP-3, MMP-13, BMP-2, IGF-1, and TGF-β1)	TLN-treated animals presented a reduction in the severity of macroscopic and histologic cartilage lesions and showed ↓MMP-1, MMP-3, and MMP-13 levels.
Moreau et al. [45]	Crossbred dog - 2–3 years old (16)	Surgically induced OA: ACLT Knee	Tiludronate (2 mg/kg/every two weeks s.c.) 6 weeks	0 days	8 weeks Macroscopic and histological grading of cartilage and synovial (OARSI scoring system) Histomorphometry (cCg.Th, SB.Th, Tb.Th, and Tb.S). Synovial fluid and inmunohistochemistry (PGE2, NOX, MMP-1, MMP-13, cathepsin K, and ADAMTS-5)	TLN treated animals having less joint effusion, lower synovitis score and a greater subchondral bone surface. ↓PGE2, NOX, MMP-13, cathepsin K and ADAMTS5. TLN failed to prevent or correct cartilage lesion and osteophyte development.

*ACLT* anterior cruciate ligament transection, *ADAMTS* a disintegrin and metalloproteinase with thrombospondin motifs, *ALN* Alendronate, *BMD* bone mineral density, *BAP* bone-specific alkaline phosphatase, *BPs* bisphosphonates, *BMP* bone morphogenic protein, *BS/BV* bone surface to bone volume ratio, *BV/TV* bone volume fraction, *CACTC* cyclic articular cartilage tibial compression, *cCg.Th* calcified cartilage thickness, *COLX* type-X collagen, *Conn.D* connectivity density, *COMP* cartilage oligomeric matrix protein, *CS846* chondroitin sulfate 846, *μ-CT* micro-computed tomography, *Ct.Th* cortical thickness, *CTX* collagen carboxyterminal telopeptide, *IGF* insulin-like growth factor, *IL* interleukin, *MCL* medial collateral ligament, *KTI* knee triad injury, *MIA* monosodium iodoacetate, *MMP* matrix metalloproteinase, *MMT* medial meniscectomy, *MRI* magnetic resonance imaging, *NOx* nitrites and nitrates, *PCR* polymerase chain reaction, *OA* osteoarthritis, *OARSI* Osteoarthritis Research Society International, *OVX* ovariectomized, *PAM* pamidronate, *PG* prostaglandin, *P1NP* procollagen type 1 N-terminal propeptide, *RANKL* receptor activator of nuclear factor-kappa B ligand, *RIS* risedronate, *SB.Th* subchondral bone thickness, *SMI* structural model index, *Tb.N* trabecular number, *Tb.Pf* trabecular bone pattern factor, *Tb.S* trabecular separation, *Tb.Th* trabecular thickness, *TGF* transforming growth factor, *TRAP* tartrate-resistant acid phosphatase, *ZLN* zoledronic acid, *VEGF* vascular endothelial growth factor, *TLN* tiludronate. *Start point: time between induced OA and treatment administration

#### Animals and osteoarthritis models

Preclinical studies included healthy animals of both sexes and were performed in rabbits (9 out of 26; 34.6%) and rats (9 out of 26; 34.6%), followed by mice (3 out of 26; 11.5%), guinea pigs (2 out of 26, 7.8%), and dogs (3 out of 26; 11.5%). In this review, in the majority of the studies that included dog and rabbit models, OA was surgically induced by anterior cruciate ligament transection (ACLT) and/or medial meniscectomy (MMT) [20–27, 43–45]. Only one study in rabbits used intraarticular injection of chymopapain for chemically-induced OA [28]. Regarding experimental rodents, several animal models were used for mimicking OA such as surgically induced models through ACLT [29, 37], MMT [30, 33], or knee triad injury (KTI) [35]; chemically-induced models by an injection of monosodium iodoacetate (MIA) [32, 36, 40], non-invasive loading models [38, 39], and spontaneous-models [31]. Lastly, we included two studies employing guinea pigs as spontaneous OA knee models [41, 42].

#### Types of bisphosphonates

Five types of bisphosphonates were analyzed. The most common type was alendronate, studied in 11 articles: two in rabbits [23, 24], five in rats [31–33, 35, 37], three in mice [38–40], and one in guinea pigs [42]. It was followed by zoledronic acid with 7 articles: three in rabbits [20, 21, 28], three in rats [29, 30, 36], and one in dogs [43], and risedronate with 6 studies: four in rabbits [22, 25–27], one in rats [35], and one in guinea pigs [41]. Two studies in dogs evaluated the effect of tiludronate [44, 45], and only one study in rats focused on the effect of pamidronate [34]. It should also be noted that in one of the studies [35], the effect of two types of BPs (alendronate and risedronate) was evaluated.

#### Therapy duration

The great majority of the studies included in this review were based on short periods of treatment administration, with ≤2 months of BP therapy (*n* = 17). Regarding intermediate periods of time, we identified 7 articles, with therapy durations between 2.5 and 4.5 months. Finally, we only identified 2 publications with drug therapies ≥6 months, one which evaluated the effect of risedronate for 6 months in guinea pigs [41], and another which used zoledronic acid for 1 year in experimental dogs [43] (Table 2).

**Table 2:** Therapy duration of bisphosphonates

Animal model	Short-term (***≤*** 2 months)			Intermediate-term (2 to 6 months)			Long term (≥6 months)		
	Reference	Drug	Duration	Reference	Drug	Duration	Reference	Drug	Duration
Rabbit	She et al. [20]	ZLN	*	Shirai et al. [23]	ALN	3			
	Lamproploulou et al. [21]	ZLN	1						
	Permuy et al. [22]	RIS	2						
	Zhang et al. [24]	ALN	2						
	MacNeil et al. [25]	RIS	1.5						
	Doschack et al. [26]	RIS	1.5						
	Doschack et al. [27]	RIS	1.5						
	Muehleman et al. [28]	ZLN	2						
Rat	Cinar et al. [29]	ZLN	1.5	Bagi et al. [30]	ZLN	2.5			
	Mohan et al. [32]	ALN	< 1	Zhu et al. [31]	ALN	4.5			
	Panahafir et al. [33]	ALN	2	Hayami et al. [37]	ALN	2.5			
	Koh et al. [34]	PAM	2						
	Strassle et al. [36]	ZLN	< 1						
	Jones et al. [35]	ALN/RIS	2						
Mice	Adebayo et al. [38] Khorasani et al. [39]	ALN ALN	1.5 1	Sniekers et al. [40]	ALN	3			
Guinea-Pig				Ding et al. [42]	ALN	4.5	Thomsen et al. [41]	RIS	6
Dog	Moreau et al. [45]	TLN	1.5	Pelletier et al. [44]	TLN	3	Dearmin et al. [43]	ZLN	12

*ALN* alendronate, *PAM* pamidronate, *RIS* risedronate, *ZLN* zoledronic acid, *TIL* tiludronate. Duration in months. *Only once post-surgery

#### Synthesis of main outcomes of the effect of bisphosphonates

Preclinical studies were evaluated based on the effect of BPs on cartilage, subchondral bone, synovial membrane, osteophyte formation, and biochemical OA markers (Table 3). The most studied parameter was the cartilage status, assessed in all publications (*n* = 26), followed by the subchondral bone (*n* = 19), the biochemical analysis (*n* = 13), and the osteophyte development (*n* = 11). The synovial assessments (*n* = 4) are ranked last.

**Table 3:** Synthesis of main outcomes of the effect of bisphosphonates

Drug	Reference	C	SB	SM	OST	BM
**Alendronate (n = 11)**	Adebayo et al. [38]	?	+	x	?	x
	Khorasani et al. [39]	?	?	x	–	+
	Zhu et al. [31]	?	+	x	x	+
	Mohan et al. [32]	?	+	x	x	?
	Panahafir et al. [33]	+	x	x	+	x
	Shirai et al., 2011 [23]	+	+	x	–	+
	Zhang et al. [24]	+	+	x	x	+
	Sniekers et al. [40]	+	+	x	x	x
	Jones et al. [35]	+	+	x	+	x
	Ding et al. [42]	–	+	x	x	x
	Hayami et al. [37]	+	+	x	+	+
**Zoledronic acid (*n*** **= 7)**	She et al. [20]	+	+	x	x	x
	Cinar et al. [29]	+	x	+	x	–
	Bagi et al. [30]	–	+	x	–	+
	Lampropoulou et al. [21]	+	x	x	x	x
	Dearmin et al., 2014 [43]	+	x	x	–	+
	Strassle et al. [36]	+	+	?	x	x
	Muehleman et al., 2002 [28]	+	x	x	x	+
**Risedronate (*n*** **= 6)**	Permuy et al. [22]	+	+	+	x	x
	Thomsen et al. [41]	–	?	x	x	+
	Jones et al. [35]	+	+	x	–	x
	MacNeil et al. [25]	+	+	x	–	x
	Doschak et al. [26]	–	+	x	x	x
	Doschak et al. [27]	–	+	x	–	x
**Tiludronate (n = 2)**	Moreau et al. [45]	–	+	+	–	+
	Pelletier et al. [44]	+	x	x	x	+
**Pamidronate (n = 1)**	Koh et al. [34]	+	+	x	x	x

*C* cartilage, *SB* subchondral bone, *SM* synovial membrane, *OST* osteophyte, *BM* biochemical markers. (+) Positive effect; (−) negative effect or no effect; (?) unclear effect; (x) not included

Overall, the cartilage was typically evaluated through gross and histologic analysis. It is seems that the zoledronic acid showed the greatest chondroprotective effect, with only one article (1 out of 7) where the drug failed to prevent or correct cartilage deterioration [30]. The use of alendronate exhibited positive effects in more than half of the studies analyzed (6 out of 11) with only one negative effect, recorded in a guinea pig study [42], while risedronate treatment showed variable efficacy, with three articles classified as negative or no effect (3 out of 6) [26, 27, 41]. Regarding the subchondral bone evaluations, most were performed by histomorphometric analysis and imaging techniques. BP-based therapy exhibited greater periarticular bone conservation and quality, and only two studies showed unclear effects, one which used alendronate [39] and another which studied the risedronate efficacy [41]. The biochemical markers of OA were mostly analyzed in serum, urine samples, and immunohistochemical assays. Out of thirteen included publications, BP-based therapy had a positive effect in eleven of them. Only one study on alendronate showed contradictory results [32], and one focusing on zoledronic acid exhibited no significant differences against the control group [29]. As far as osteophyte development is concerned, BPs were not able to inhibit the osteophytogenesis in most cases (9 out of 11), and only alendronate therapy showed an inhibitory response in three of the studies analyzed [33, 35, 46]. Lastly, the synovial inflammation was evaluated only in four studies, two focusing on zoledronic acid [29, 36], one on risedronate [22], and another on tiludronate [45]. Overall, BP treatments seemed to lower synovitis scores showing a tendency toward anti-inflammatory effects.

### Quality and risk of bias assessments

#### Quality assessments

**Fig. 2:**
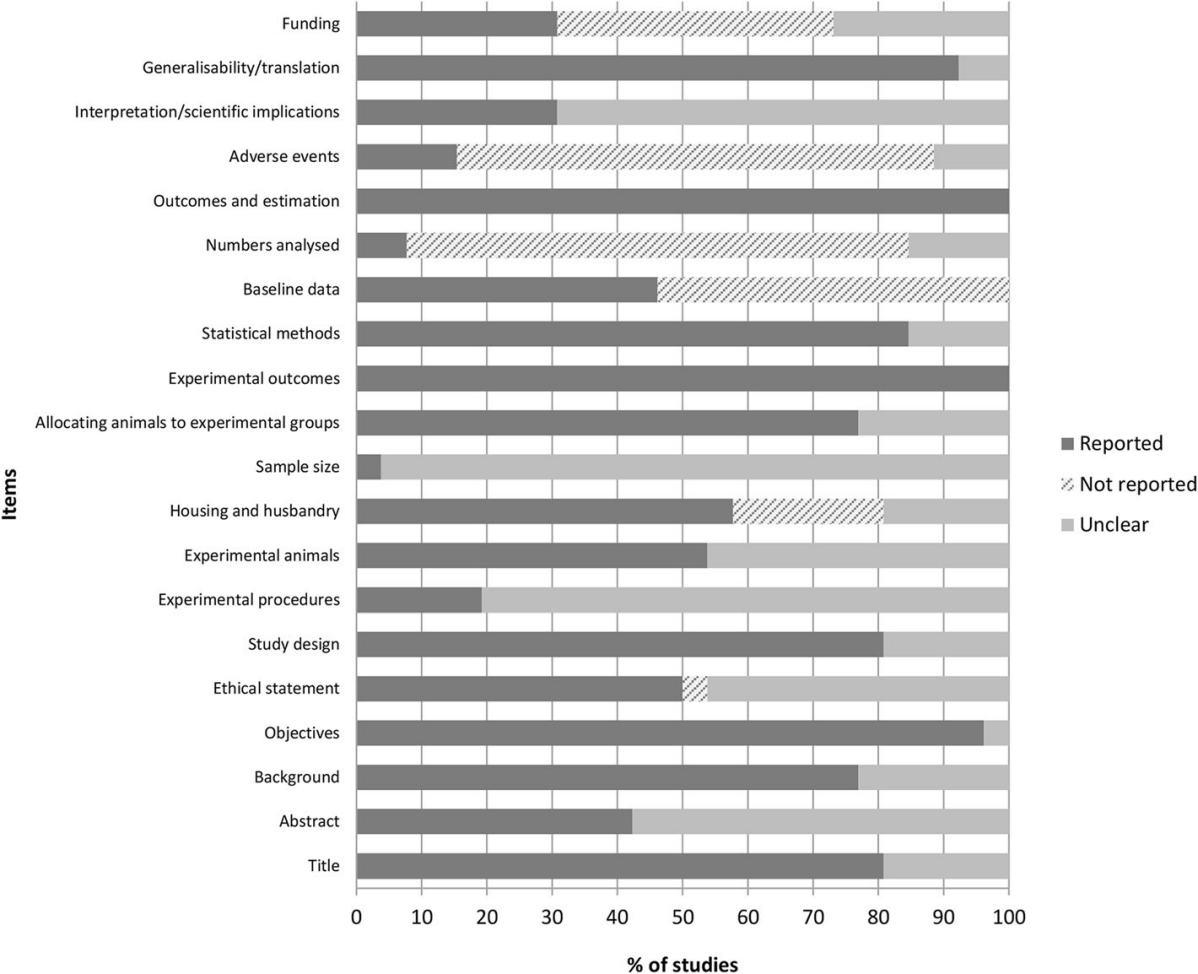
Quality assessments of the preclinical studies based on the Animals in Research Reporting In Vivo Experiments (ARRIVE) guidelines. Values are expressed by frequencies (%)

Figure 2 summarizes the quality assessments of the preclinical studies based on the ARRIVE guidelines. The results showed that in most papers at items 9 “Housing and husbandry, 14 “Baseline data”, 15 “Number analysed, 17 “Adverse events”, and 20 “Funding,” an evident lack of information was observed, with 23.1%, 53.85%, 76.9%, 73.1%, and 42.3% of frequencies respectively classified as “not reported.” By contrast, items 1 “Title,” 3 “Background,” 4 “Objectives,” 6 “Study design,” 11 “Allocating animals to experimental groups,” 12 “Experimental outcomes,” 13 “Statistical methods, 16 Outcomes and estimation,” and 19 “Generalisability/translation” were classified as “reported,” showing high percentages of completed items, with frequencies of 80.8%, 76.9%, 96.15%, 80.8%, 76.9%, 100%, 84.6%, 100%, and 92.3%. The remaining items, 2 “Abstract,” 5 “Ethical statement,” 7 “Experimental procedures,” 8 “Experimental animals,” 10 “Sample size,” and 18 “Interpretation/scientific implications,” were assigned as “unclear,” showed incomplete items or did not report any sub-items, with frequencies of 57.7%, 46.15%, 80.8%, 46.15%, 96.15%, and 69.2%.

#### Risk of bias

**Fig. 3:**
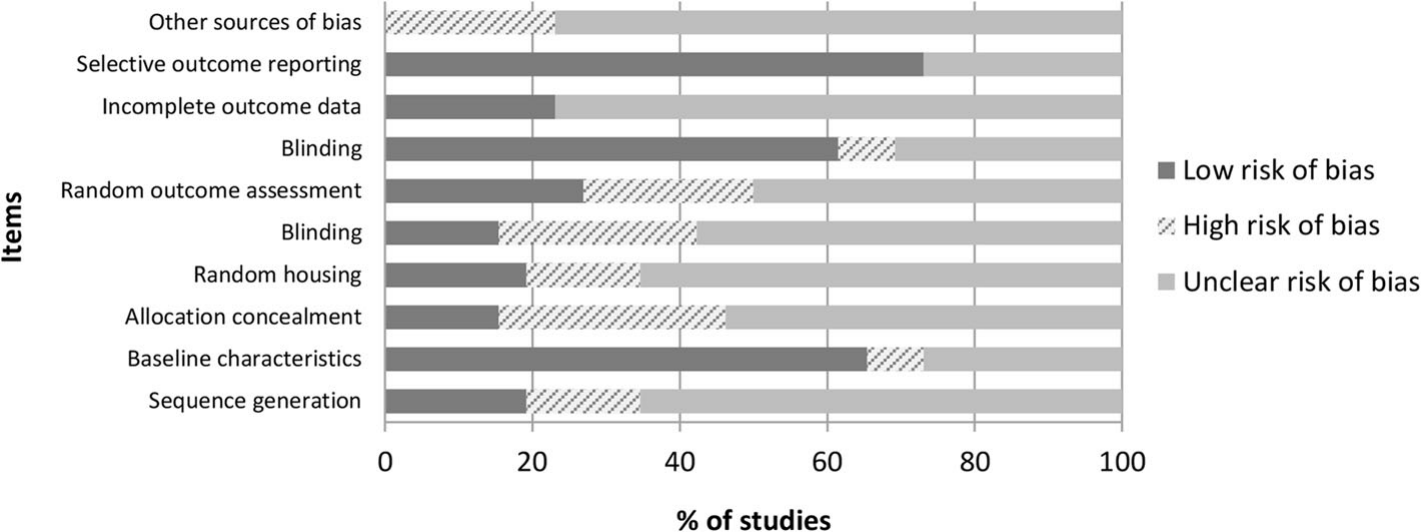
Risk of bias distribution graph according to Systematic Review Centre for Laboratory animal Experimentation (SYRCLE) tool. Values are expressed by frequencies (%)

The graphic results of the risk of bias assessments in the included studies were shown in Fig. 3 using the SYRCLE tool. Generally, most of the items evaluated were assigned as “unclear risk of bias” showing frequencies above 50%. The higher risk of bias was observed at items 3 “Allocation concealment,” 5 “Blinding of caregivers and/or investigators,” 6 “Random outcome assessment,” and 10 “Other sources of bias,” with frequencies of 30.8%, 26.9%, 23.1%, and 23.1%, respectively. By contrast, the lower risk of bias was assigned at items 2 “Baseline characteristics,” 7 “Blinding of outcome assessor,” and 9 “Selective outcome reporting,” with frequencies of 65.4%, 61.5%, and 73.1%.

## Discussion

The aim of this systematic review was to study the effect of bisphosphonates in osteoarthritic experimental animal models. In this study, a total of 26 publications with 5 different types of BPs were selected in order to elucidate whether these antiresorptive therapies could somehow influence the OA progression. To the best of our knowledge, no previous systematic reviews have evaluated their effect on synovial joint tissues and biochemical markers in preclinical models. However, a literature review analyzing the uses of BPs as a treatment modality in OA preclinical and clinical studies has been recently published concluding that there are some appreciable discrepancies between them [47]. Regarding human studies, we only identified one meta-analysis which examined the efficacy of BPs in the treatment of OA pain in humans [15] and other two which analyzed the effects of BPs compared with placebo in terms of clinical and structural outcomes in specific human knee OA [13, 14]. Even though their main results did not seem to be very promising, some of them suggested that their administration in specific patients with high rates of subchondral bone turnover may be beneficial [13, 14].

Although in some cases translating preclinical findings into clinical management of OA may not be highly reliable, animal preclinical models may provide an excellent opportunity to evaluate the direct effect of different therapies on affected joints [48]. In this review, the most commonly used animal models were rabbits and rats. Among their main advantages, one should point out their easy management and low maintenance costs, surgeries are easier to perform than in smaller animals such as mice, complete genomes are available from study, and both are useful in studying the efficacy of compounds [49]. Other animal models as mice, guinea pigs, and dogs were also included in this study. It is important to highlight that the variety of animal species and the different models of OA could make direct comparison among studies challenging. Moreover, it is important to underline that no single animal model is able to mirror all variants and aspects of OA. Therefore, depending on our experimental goal, appropriate animal model should be chosen [12]. More than half of the publications included in this study were surgically induced models (*n* = 16), in which ACLT (*n* = 10) was the most used surgery technique. This was followed by chemically induced models (*n* = 5) which received an injection of MIA, by spontaneous models due to naturally occurring OA (*n* = 3) and finally, by non-invasive loading models (*n* = 2). These findings slightly differ to those described by other authors of OA preclinical studies. Half of these used spontaneous models, followed by surgical induction models and chemically induced models [50].

In addition to the different animal model used, various experimental protocols were reported in relation to the type of bisphosphonate, the posology, and the route of administration. Another major concern with BPs is that the bioavailability may be different in relation to the animal species under study, so it should be taken into account when the experiment is designed [24]. Besides, there are also notable differences among the duration and the baseline of therapy. Consequently, comparing findings in order to draw significant conclusions about the drug efficacy is challenging.

Regarding the therapy duration, we observed in the studies that currently there is a lack of evidence to evaluate their efficacy as disease-modifying drugs on the long-term. As we could observe in this review, there are almost literally no studies that evaluated the bisphosphonate effect after 6 months of treatment. In this context, we should take into account that the long-term bisphosphonate pharmacological response in preclinical studies is basically unknown. It is possible that the initial chondroprotective effect observed in some publications could not be sustained over time. Interestingly, in one of the included studies, the alendronate use was analyzed comparing different treatment durations (7, 14, or 56 days). Although all of them were based on short periods of time, differences were already observed and, in spite of a positive initial response, alendronate use was not able to mitigate the long-term OA progression [39]. Similarly, another study using guinea pigs as animal model exposed that risedronate did not have the ability to prevent articular cartilage damage after 6 months of treatment [41]. On the contrary, Dearmin et al. determined the zoledronic acid effect in a dog experimental model for a year, showing a beneficial response with less articular damage and fewer biomarkers changes [43]. Regarding clinical studies, an interesting human trial conducted for 2 years, concluded that risedronate did not have the ability to reduce the OA evolution. However, reduced cartilage degradation markers were detected [51]. Another 2-year clinical trial on hip OA showed that osteoporosis-standard dosing of alendronate (35 mg/week) did not demonstrate an evident disease-modifying effect, but decreased clinical pain and showed lower C-telopeptide fragments of type II collagen (CTX-II) levels [52]. According to this, it is interesting to observe that for an optimal response on cartilage protection, higher BP doses than those used to treat osteoporosis may be required. Among the studies included in this review, 5 out of 26 publications studied the efficacy of these therapies at different doses. Generally, they concluded that BPs seemed to reduce the osteoarthritic changes in a dose-dependent manner showing better chondroprotective effects at high doses [20, 36, 37, 39, 43].

Another point of interest is the treatment timing initiation in relation to OA stage. It has been observed that pre-emptive and early BPs therapies may lead to improved outcomes. By contrary, delayed treatments have been associated with reduced chondroprotective efficacy. In this case, 3 experimental studies evaluated the alendronate or zoledronic acid effects on cartilage degradation and subchondral bone quality at various points of OA stage [31, 32, 36]. Although certain positive effects were observed on the subchondral bone, independently of the time point of treatment initiation, an obvious time-dependent efficacy was detected on cartilage status. As proposed by Strassle et al., these findings may partially explain the differences in outcomes observed between clinically and preclinically studies. Given that some positive findings reported in experimental studies could be related to initial uses of BPs in early stages of OA, while in clinical trials, therapies are usually initiated when the disease is advanced [36]. For this reasons, we can conclude that the stage of disease when treatments are employed is a key factor in the effectiveness of the antiresorptive therapies.

Among this high experimental variability, it is extremely difficult to draw specific conclusions regarding the effectiveness of BPs. Generally, in this review, we noted that antiresorptive administration seemed to show positive subchondral bone conservation and fewer biomarker alterations. However, they did not appear to suppress the osteophyte formation. Regarding the cartilage status, the observed effects of BPs varied among the studies but overall, zoledronic acid exhibited the greatest chondroprotective response [20, 21, 28, 29, 36, 43]. Additionally, there is no consensus on which are the most adequate methods of evaluation. As previously discussed, the parameters most commonly assessed in the preclinical studies analyzed herein were the cartilage degradation and the subchondral bone changes. It is well known that the osteoarthritis pathology involves all tissues included in the synovial joint. Moreover, synovitis has been correlated with the progression of the OA and it is the main cause of pain [53]. However, only 4 publications in this systematic review focused on the synovial inflammation [22, 29, 36, 45]. Considering that antiresorptive therapies appear to show a positive anti-inflammatory effect, further studies, including on the evaluation of the synovial membrane status, are needed in order clarify its role in the pathophysiology of the disease.

Regarding the quality evaluation of the preclinical animal studies included in this review, most of the publications showed quite well reported items. Nevertheless, there were several key items that we identified as poorly reported, similarly to what was observed in other reviews of preclinical OA studies [50]. Half of the publications included showed incomplete ethical statements with lack of information in relation to animal care guidelines. Besides, the majority of studies did not provide precise details about anesthesia and analgesia protocols, the method of euthanasia, or further relevant information such as the source of animals. Incomplete details about housing and husbandry were reported in almost half of the studies. In terms of sample size, only one study explained how the number of animals was arrived at [38]. This observation coincided with that observed in other systematic review with animal studies in rheumatology, since this item was not reported in any paper [54]. Additionally, we observed an important lack of details such as the health status description, the absolute number of animals included, and the description of adverse events, once more in agreement with Ting et al. In relation to assessments of risk of bias, as reported in other systematic reviews with animal studies, we observed an appropriate description of baseline characteristics and selective outcome reporting [55]. The same applied to the blinding of outcome assessor [50]. By contrast, a higher risk of bias was identified at allocation concealment, the randomly housed animals during the experiment, and the blinding of caregivers [55]. Additionally, many entries had to be judged as “unclear risk of bias” showing that some research publications on animals fail to report important information. An interesting previous survey research of 271 animals studies revealed that reported experimental details on animals, methods, and materials continue to be very poor [56]. According to this, we consider improving the reporting quality of essential details in experimental animal studies is essential [19].

Our study has several limitations to consider. One of them is that this study did not include a meta-analysis. Another limitation is the great heterogeneity in the variables of the experimental studies, which make comparisons among publications challenging. Additionally, the quality and methodology of the experimental animal studies was highly variable.

## Conclusions

Bisphosphonates have been proposed as possible disease-modifying drugs in OA, but as far as published preclinical studies are concerned, they show a great heterogeneity in their outcomes. Additionally, the evidence of their efficacy is poor and, at present, hardly any long-term studies have been conducted. In this review, significant differences were observed in the experimental designs, including the variety of OA animal models and the drug type, duration, and posology. Consequently, it is extremely difficult to draw specific conclusions about the effectiveness of these drugs. However, the results of this systematic review suggested that the type of dose selected and the time point of treatment initiation may be two key factors in the effectiveness of these therapies, highlighting better chondroprotective effects at high doses and pre-emptive administrations. Regarding the therapy duration, long-term studies are needed to elucidate the effect of BPs over time. Additionally, we noted that antiresorptive administration seemed to improve the subchondral bone quality and show fewer biomarker alterations. However, they did not appear to supress the osteophyte development and their chondroprotective effect is highly variable among the studies. Lastly, with reference to synovial membrane evaluation, bisphosphonate treatments seemed to show a tendency toward anti-inflammatory effect but further studies are needed in order to clarify their effectiveness.

## Data Availability

Data sharing is not applicable to this article as no datasets were generated or analyzed during the current study.

## Abbreviations

ACLT: Anterior cruciate ligament transection
ALN: Alendronate
ARRIVE: Animals in Research Reporting In Vivo Experiments
BPs: Bisphosphonates
CTX-II: C-telopeptide fragments of type II collagen
KTI: Knee triad injury
MIA: Monosodium iodoacetate
MMT: Medial meniscectomy
OA: Osteoarthritis
PAM: Pamidronate
PRISMA: Preferred Reporting Items for Systematic Reviews and Meta-Analyses
RIS: Risedronate
SYRCLE: Systematic Review Centre for Laboratory animal Experimentation
TIL: Tiludronate
WOS: Web of Science
ZOL: Zoledronic acid
